# Preparation and Characterization of Protein Molecularly Imprinted Poly (Ionic Liquid)/Calcium Alginate Composite Cryogel Membrane with High Mechanical Strength for the Separation of Bovine Serum Albumin

**DOI:** 10.3390/molecules27217304

**Published:** 2022-10-27

**Authors:** Jie-Ping Fan, Wen-Ya Dong, Xue-Hong Zhang, Jia-Xin Yu, Cong-Bo Huang, Li-Juan Deng, Hui-Ping Chen, Hai-Long Peng

**Affiliations:** 1 Department of Chemical Engineering, Nanchang University, Nanchang 330031, China; 2 School of Foreign Language, Nanchang University, Nanchang 330031, China; 3 School of Chemical Engineering, Ningbo University of Technology, Ningbo 315016, China

**Keywords:** molecularly imprinted cryogel membrane, poly (ionic liquid), alginate, separation, bovine serum albumin

## Abstract

In order to improve the mechanical strength and imprinting efficiency, a novel bovine serum albumin (BSA) molecularly imprinted poly(ionic liquid)/calcium alginate composite cryogel membrane (MICM) was prepared. The results of the tensile test indicated that the MICM had excellent mechanical strength which could reach up to 90.00 KPa, 30.30 times higher than the poly (ionic liquid) membrane without calcium alginate; the elongation of it could reach up to 93.70%, 8.28 times higher than the poly (ionic liquid) membrane without calcium alginate. The MICM had a very high welling ratio of 1026.56% and macropore porosity of 62.29%, which can provide effective mass transport of proteins. More remarkably, it had a very high adsorption capacity of 485.87 mg g^−1^ at 20 °C and 0.66 mg mL^−1^ of the initial concentration of BSA. Moreover, MICM also had good selective and competitive recognition toward BSA, exhibiting potential utility in protein separation. This work can provide a potential method to prepare the protein-imprinted cryogel membrane with both high mechanical strength and imprinting efficiency.

## 1. Introduction

Molecular imprinting technology is a synthetic method for preparing polymer materials with a memory of the size, shape and structure and functional groups of the selected template molecules. This technology has the characteristics of high specific recognition, a wide application range and a predictable synthesis process [1,2,3]. At present, molecular imprinting technology has been used widely in many fields [4,5,6,7,8], especially in the field of separations [9,10,11,12,13]. However, the development of protein molecular imprinting technology still confronts many challenges, mainly because of the intrinsic properties of proteins, such as the large size, structural variability, complex surface groups and easy denaturation and unfolding during the separation process [14,15]. To solve these problems, the molecularly imprinted hydrogel is a good choice, because the biocompatibility of the hydrogel-based system does not affect protein conformation and the macroporous structure of hydrogel can act as the passages of the proteins [15,16,17]. What is especially worth noting is that supermacropores can be formed in cryogels during frozen polymerization, which can allow a rapider and non-restricted mass-transport of proteins than common hydrogels [18,19]. Moreover, it is believed that the functional ionic liquid (IL) is a high-performance monomer for preparing protein molecularly imprinted polymer (MIP) because IL is designable and can provide multiple interactions and active sites [15,17,18,19]. Compared with traditional functional monomers, ILs can interact with the target molecules through multiple bonds, such as ion-pair interaction, hydrogen bond and π-π interaction. Therefore, IL is a good candidate as the functional monomer to improve the performance of MIP [17,18,19,20]. However, we found that the traditional poly (ionic liquid) (poly(IL))-based hydrogels had poor mechanical properties, usually showing low-stress strength, poor toughness and low elongation [15], because of their high water contents and relatively fragile polymer networks in the fully swollen state. The poor mechanical stability will limit the separation efficiency and the reusability of MIP. To enhance the mechanical strength, adding another component has been attempted [16,21,22,23,24], because the good mechanical property of the composite constructs can maintain the shape of the imprinted cavities to improve the regeneration properties of the hydrogels [22,23].

**Scheme 1:**
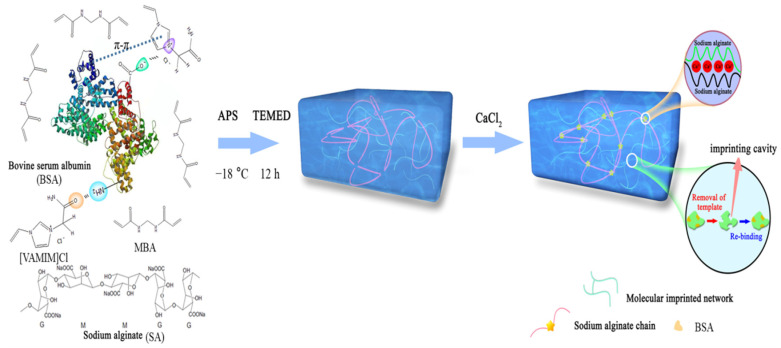
Schematic diagram of the preparation process of the MICM.

Herein, we reported a method for preparing bovine serum albumin (BSA) molecularly imprinted poly(IL)/calcium alginate (CA) composite cryogel membrane (MICM). In the synthesis process, we used a specific ionic liquid 1-vinyl-3-carbamoyl imidazole chloride ([VAMIM]Cl) as the functional monomer and N,N′-methylenebisacrylamide (MBA) as the covalent cross-linking agent, and their chemical structures were listed in Scheme 1. In this work, [VAMIM]Cl can interact with BSA through the imidazole ring and amide group to form multiple interactions. Sodium alginate (SA) is very suitable for preparing protein MIP because of its good biocompatibility, natural degradability, strong water solubility and non-toxic properties [22,23,24]; moreover, SA can also interact with the protein to improve the imprinting efficiency. After the radical polymerization of ILs in the phosphate buffer solution (PBS, pH = 7.0), SA was ionically cross-linked by calcium ions (Ca^2+^) to form calcium alginate (CA) chain. As shown in Scheme 1, MICM consisted of the covalently cross-linked poly(IL) and CA network structures, which can enhance the mechanical property; moreover, the poly(IL) and CA can improve the interaction between BSA and the membrane. Therefore, it was expected that MICM would have both high mechanical strength and separation efficiency. To the best of our knowledge, a BSA molecularly imprinted poly(IL)/CA composite cryogel membrane has not been reported yet. It should be noted that in this work BSA was only chosen as a model protein, and we think the method can also be used for the synthesis and application of other protein-imprinted polymers.

## 2. Results and Discussion

### 2.1. Characterization of the Membrane

**Figure 1:**
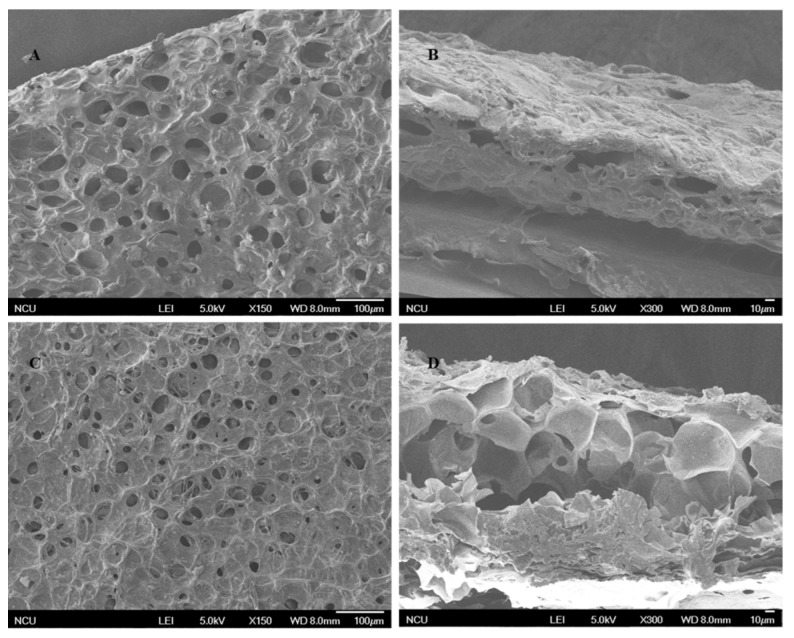
SEM images of the surface (**A**,**C**) and cross-section (**B**,**D**) of MICM (**A**,**B**) and NMICM (**C**,**D**).

As shown in Figure 1, the MICM and NMICM had a large number of supermacropores with several micrometers in size because of ice crystals as the pore templates during the frozen polymerization, which was beneficial for improving mass transfer and adsorption capacity.

**Figure 2:**
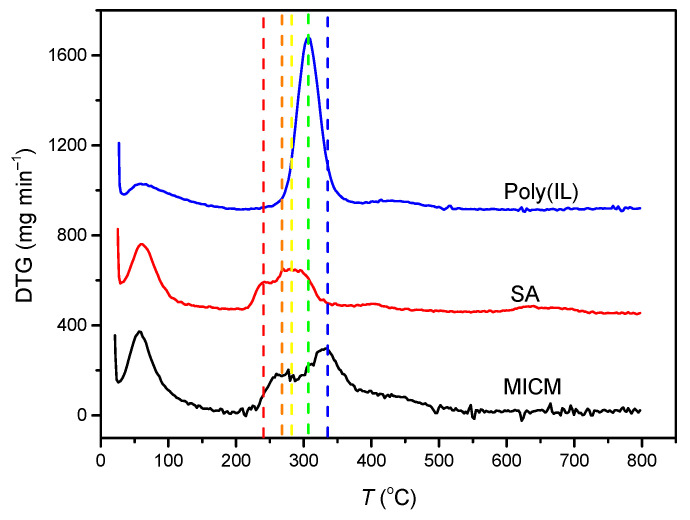
Differential thermal gravimetric (DTG) analysis of the membranes.

Figure 2 showed the DTG curves of poly(IL), SA and MICM. The maximum thermal degradation rate of poly(IL) occurred at 307 °C, the DTG peak of SA was at 240 and 285 °C, and the DTG peak of MICM was at 267 and 335 °C. Compared with the DTG peak of poly(IL) and SA, the DTG peak of MICM shifted to higher temperatures, which indicated that the poly(IL) and SA had formed the composite structure, which could enhance the thermal stability of MICM.

**Figure 3:**
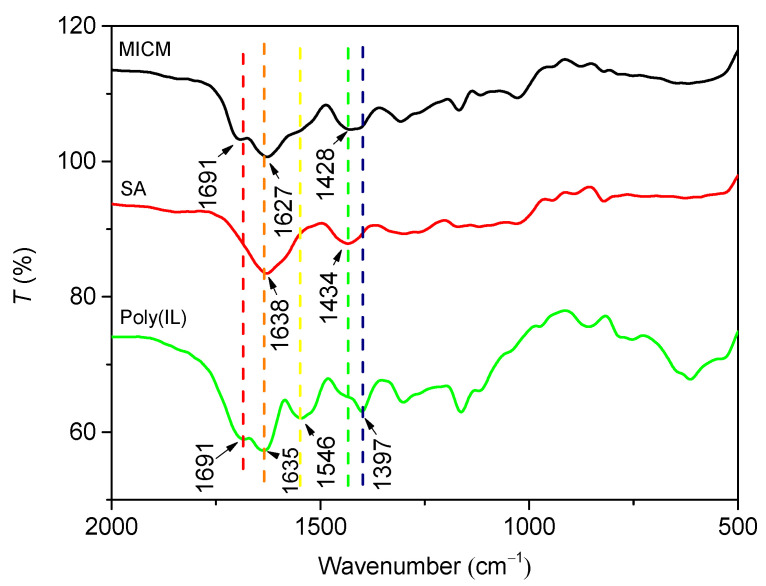
FT-IR spectra of MICM, SA and poly(IL).

In Figure 3, the two characteristic absorption peaks of SA at 1638 and 1434 cm^−1^ were attributed to asymmetric and symmetric stretching of the carboxylate group −COO−, respectively [25]. The peak of poly(IL) at 1691 cm^−1^ was attributed to C = O stretching vibration; the peak at 1546 cm^−1^ was assigned to the skeleton vibration of the imidazole ring of the IL; and the peaks at 1397 cm^−1^ was due to the stretching vibration C−N [26]. The infrared spectrum MICM was the rough superposition of the infrared spectra of CA and poly(IL), whereas the peak of −COO− stretching vibration shifted to a lower wavenumber, which indicated that the poly(IL)/CA composite structure has formed [27].

**Figure 4:**
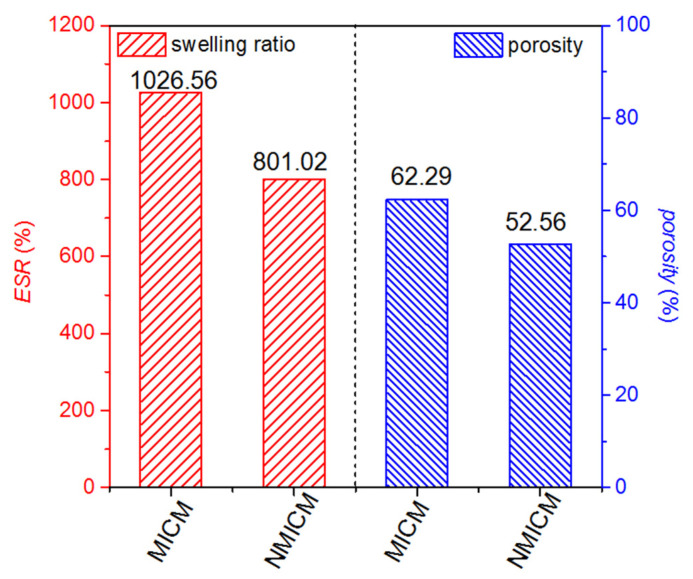
*ESR* and *porosity* of MICM and NMICM.

The *ESR* and *porosity* of MICM and NMICM were determined at the equilibrium time by the weighing method and plotted in Figure 4. Both *ESR* and *porosity* of MICM were higher than those of NMICM because the removal of BSA from MICM would cause more gaps and pores in the cross-linked polymer chain. The higher macropore *porosity* of MICM was beneficial to the mass-transport of protein.

### 2.2. Optimization of Synthesis Conditions of MICM

**Figure 5:**
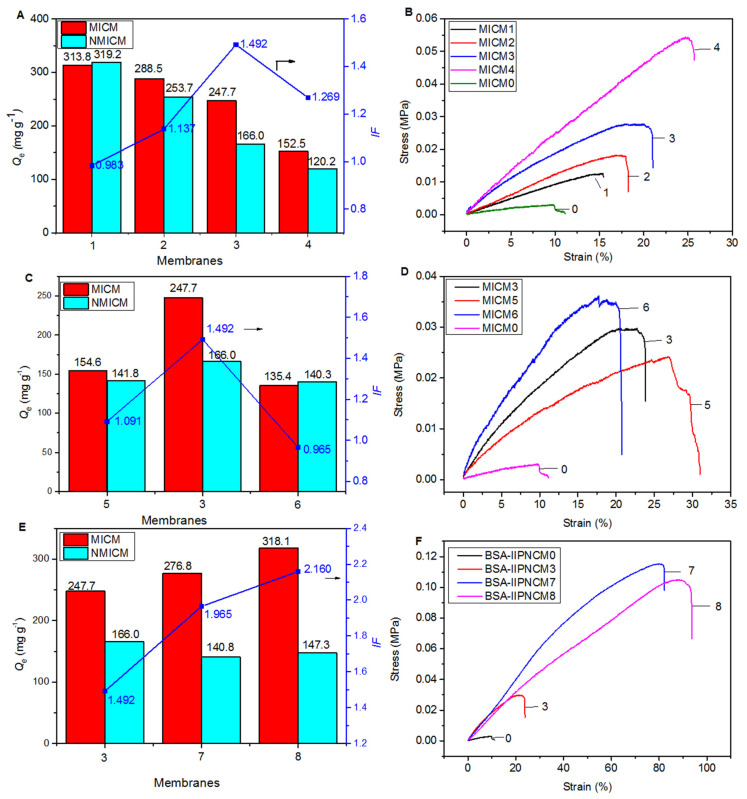
The effects of the amount of SA, cross-linker, [VAMIM]Cl and MBA on the adsorption (**A**,**C**,**E**) and mechanical (**B**,**D**,**F**) properties of various MICM and NMICM.

To optimize the synthesis conditions of MICM, the effects of the amount of SA, [VAMIM]Cl and MBA on the *Q_e_*, *IF* and mechanical strength of the membrane were investigated. The experimental conditions and results are listed in Table S1 and Figure 5. 

First, the effect of the amount of SA on the adsorption (Figure 5A) and mechanical (Figure 5B) properties were studied, and the membranes (MICM0-4) were prepared according to the experimental conditions in Table S1. The results of MICM0 were cited from our previous work [15]. As shown in Table S1, Figure 5A,B, MICM0 had the best adsorption performance among the five MICMs, but the mechanical strength was the lowest. Therefore, the formation of a composite structure could significantly improve the mechanical strength which increased with the amount of SA; however, *Q_e_* decreased with the amount of it. The results indicated that SA was very important to improve the mechanical strength, while the poly(IL) was useful for maintaining good adsorption performance. For MICM3, IF was the highest among the four MICMs (MICM1-4), and relatively high *Q_e_* and mechanical strength were achieved.

Second, the effect of the amount of cross-linker (MBA) was investigated on the adsorption (Figure 5C) and mechanical (Figure 5D) properties of the membranes (MICM 3, 5, 6). In Figure 5C and Table S1, it was found that too much or too little cross-linker would reduce the adsorption amount and the imprinting efficiency. This may be because when the amount of MBA was too small, the structure of the polymer was not stable enough to maintain the integrity of the imprinted cavity, leading to a decrease in the adsorption amount and imprinting efficiency. When the amount of MBA was too large, the pores of the polymer were too small, which was not conducive to protein transfer. As shown in Figure 5D, as the amount of the cross-linker increased, the tensile strength of the membrane would increase but the breaking elongation would decrease, because the increase in cross-linking could improve the tensile strength but reduce the stretchability of the polymer. Compared with MICM5, the tensile strength of MICM6 increased from 26.9 KPa to 30.83 KPa, but the breaking elongation decreased from 30.83% to 20.44%. 

Third, by changing the amount of [VAMIM]Cl and MBA, we investigated the effect of the amount of poly(IL) in the composite structure on the adsorption (Figure 5E) and mechanical (Figure 5F) properties of the membranes (MICM 3, 7, 8). In the experiment, the mass ratio of [VAMIM]Cl to MBA was unchanged. The results in Figure 5E showed that reducing the amount of poly(IL) could improve the adsorption performance of the membrane. The reason for this phenomenon may be that reducing the amount of the poly(IL) network system would make room for the diffusion of BSA, and the carboxyl group on SA could also be used as the functional monomer to form the specific adsorption binding sites for BSA. As shown in Figure 5F, as the amount of the poly(IL) network decreased, the mechanical properties of the membrane increased because the space occupied by the rigid poly(IL) network frame decreased to provide space for the SA flexible network. Compared with MICM3, the tensile strength of MICM8 increased from 29.62 KPa to 90 KPa, and the breaking elongation increased from 23.81% to 93.7%.

After optimization, overall the MICM8 had better adsorption and mechanical performance than the other membranes, and the optimized preparation conditions were listed in Table S1. 

### 2.3. Static Adsorption Kinetics

**Figure 6:**
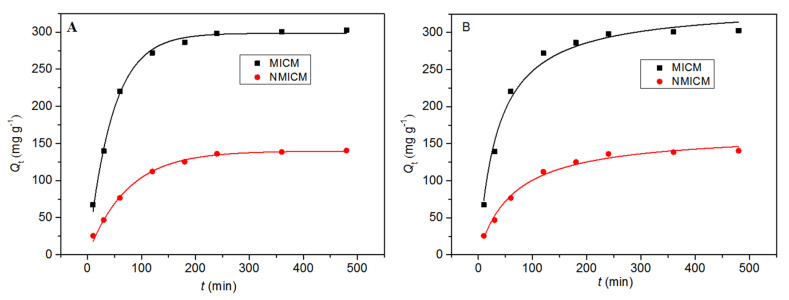
The adsorption kinetic data and fitting curves by the pseudo-first-order model (**A**) and pseudo-second-order model (**B**) at 25 °C for MICM and NMICM.

In the adsorption kinetic experiment, 0.01 g of dry membrane was fully swelled and then oscillated in 20 mL of 0.5 mg mL^−1^ BSA solution. As shown in Figure 6, the adsorption rate was relatively fast within the first 180 min, and then gradually decreased. After 360 min, the adsorption reached saturation. After equilibrium, the adsorption capacity of MICM was almost twice higher than that of NMICM, which indicated that the MICM had specific binding sites and imprinting capability for BSA. 

The adsorption data were fitted by the pseudo-first-order kinetic equation (Equation (1)) and pseudo-second-order kinetic equation (Equation (2)), [28,29,30] and the fitting curves and parameters were presented in Figure 6 and Table 1, respectively.

(1) $$Q_{t}=\frac{Q_{e}{}_{,cal}}{1-e^{-k_{1}t}}$$

(2) $$Q_{t}=\frac{k_{2}Q_{e,cal}^{2}t}{1+k_{2}Q_{e,cal}t}$$

where *Q_t_* (mg g^−1^) is the amount of protein bound on the membrane at time *t*; *Q_e,cal_* is the adsorption capacity calculated from the kinetic model; *k*_1_ and *k*_2_ are the adsorption rate constants of the pseudo-first-order and pseudo-second-order model, respectively.

In Table 1, the correlation coefficient (R^2^) of the first-order kinetic equation was higher than that of the second-order kinetic equation, and moreover, the *Q_e,cal_* value of the pseudo-first-order model was more consistent with the experimental *Q_e value_* (*Q_e,MICM_*: 302.69 mg g^−1^ and *Q_e,NMICM_*: 140.43 mg g^−1^), which suggested that the adsorption process was more suitable for the first-order kinetic equation. 

### 2.4. Static Adsorption Isotherms

**Figure 7:**
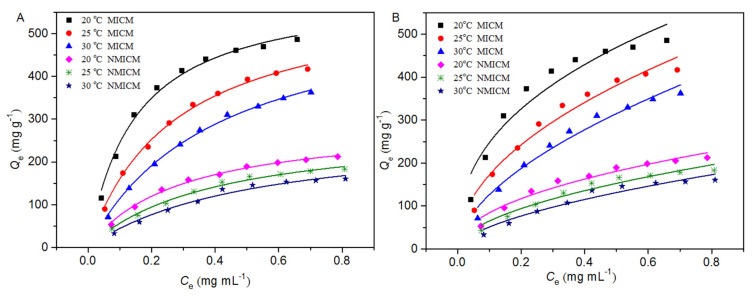
Adsorption isotherm data and the fitting curves by the Langmuir model (**A**) and Freundlich model (**B**) at different temperatures.

For the isothermal adsorption experiment, the membrane (0.01 g) was oscillated in 20 mL of BSA solution with different initial concentrations at different temperatures. In Figure 7 the isothermal adsorption data were plotted and fitted by the Langmuir model (Equation (3)) and Freundlich model (Equation (4)) [29,30,31], and the fitting curves and parameters were presented in Figure 7 and Table 2, respectively.

(3) $$Q_{e}=\frac{K_{L}Q_{max}C_{e}}{1+K_{L}C_{e}}$$

(4) $$Q_{e}=K_{F}C_{e}^{1/n}$$

where *Q_e_* and *Q_max_* (mg g^−1^) are the amount of BSA adsorbed on the membrane at adsorption equilibrium and the theoretical maximum amount of adsorption, respectively. *C_e_* (mg mL^−1^) is the concentration of BSA at adsorption equilibrium. *K_L_* (mL mg^−1^) is the Langmuir constant related to the affinity of the adsorption site. *K_F_* and n are Freundlich constants related to adsorption capacity and adsorption suitability, respectively.

To better describe the basic characteristics of adsorption capacity, a dimensionless constant (*R_L_*), derived from the Langmuir model, can be defined by Equation (5) [29].

(5) $$R_{L}=\frac{1}{1+K_{L}C_{0}}$$

where *K_L_* (mL mg^−1^) and *C*_0_ (mg mL^−1^) are the Langmuir constant and the initial concentration of BSA, respectively.

As shown in Figure 7, the equilibrium adsorption capacity of the membranes increased with the initial concentration of BSA. At the same initial BSA concentration and the same temperature, the adsorption capacity of MICM was much higher than that of NMICM because MICM had a large number of specific cavities complementary to BSA in size and shape, while the adsorption capacity of NMICM mainly resulted from non-specific adsorption. At the same initial concentration of BSA, the higher the temperature was, the lower the adsorption capacity of the membranes was, which indicated that the adsorption process was exothermic in nature. The thickness of the boundary layer decreased with temperature, which would increase the tendency of the BSA molecules to escape from the adsorbent surface to the solution phase [32]. Therefore, the adsorption capacity would decrease as the temperature increased.

In Table 2, the Langmuir model for both MICM and NMICM had higher correlation coefficients (R^2^) than the Freundlich model, which suggested that the Langmuir model was more suitable for fitting the adsorption data than the Freundlich model. According to the Langmuir model assumption, the adsorption process of the membranes belonged to the monolayer adsorption with identical adsorption sites. The values of *R_L_* were within the range of 0~1, which indicated that the adsorption processes were favorable.

### 2.5. Selective and Competitive Recognition

To evaluate the selective recognition toward BSA on the MICM, OVA, BHb and Lys were used as reference proteins. The adsorption capacity of MICM and NMICM was measured separately in these protein solutions with the same initial concentrations of 0.5 mg mL^−1^/0.8% CaCl_2_ solution. As shown in Table 3, the adsorption capacity of MICM toward BSA was much higher than that toward the other three proteins, indicating that MICM had a good selective recognition toward BSA. Moreover, the adsorption capacity of MICM toward BSA was much higher than that of NMICM. The results illustrated the MICM had higher adsorption capacity and selectivity, which can be explained by the following reasons: (1) As shown in Scheme 1, the specific imprinted cavities in the MICM had the memory of the shape, size and functional groups of BSA due to the imprinting characteristics, which would improve the selectivity; (2) the functional monomer [VAMIM]Cl can interact with BSA through multiple bonds, such as ion-pair interaction, hydrogen bond and π-π interaction, which would improve the adsorption capacity and selectivity; (3) the porous structure of the membrane facilitated the mass transfer of BSA, and would increase the adsorption capacity. For both MICM and NMICM, the adsorption capacity toward Lyz was much lower than that toward BHb and OVA, which could be explained as follows: under the experiment conditions (pH 7.0), BHb (pI 6.8) and OVA (pI 4.7) were negatively charged, while Lyz (pI 11.2) was positively charged; the membranes were positively charged due to poly(IL); therefore, the adsorption capacity toward Lyz was the lowest among these proteins due to the repulsive effect between the membrane and Lyz. 

To further confirm the imprinting efficiency of MICM toward BSA, the competitive adsorption test was carried out in the BSA/BHb mixed solution. BHb was selected as the competing protein because it had a similar molecular weight to BSA, and the *α* value for BHb was the highest (2.04) as described in the recognition selectivity experiment. The results in Table 4 showed that MICM still had good imprinting efficiency in the competitive adsorption experiment, although the α value was lower than that in the selective recognition experiment due to the competitive adsorption of BSA and BHb on the membrane.

In Table 5, the maximum adsorption capacity of some different adsorbents for BSA was compared. Compared with other adsorbents, the MICM in this work had a larger BSA adsorption capacity due to the multiple interactions including ion-pair interaction, hydrogen bond, π-π interaction and other non-covalent bonding.

### 2.6. Reusability

**Figure 8:**
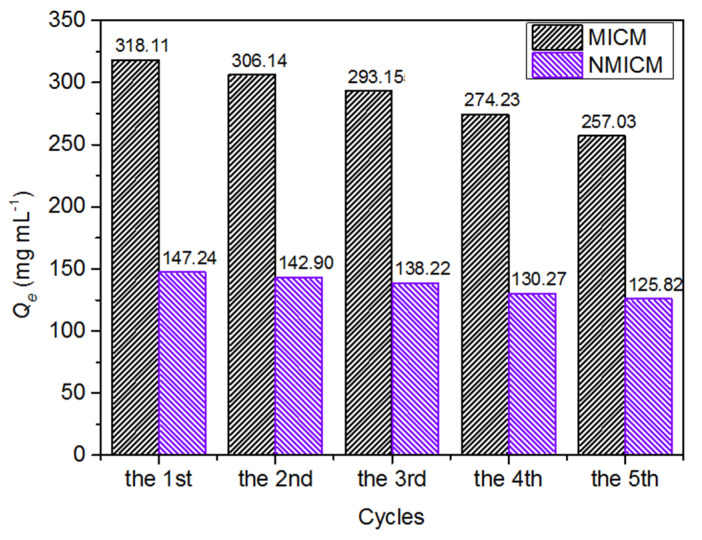
Reusability of MICM and NMICM.

As shown in Figure 8, the adsorption capacity of MICM and NMICM slightly decreased with the reuse cycles because some adsorption sites were destroyed during the reuse, while the adsorption capacity of MICM still remained at 80.8% of the initial value, which indicated that the MICM had good reusability and good stability during several adsorption–desorption cycles.

### 2.7. Membrane Permeation and Dead-End Filtration Performance

**Figure 9:**
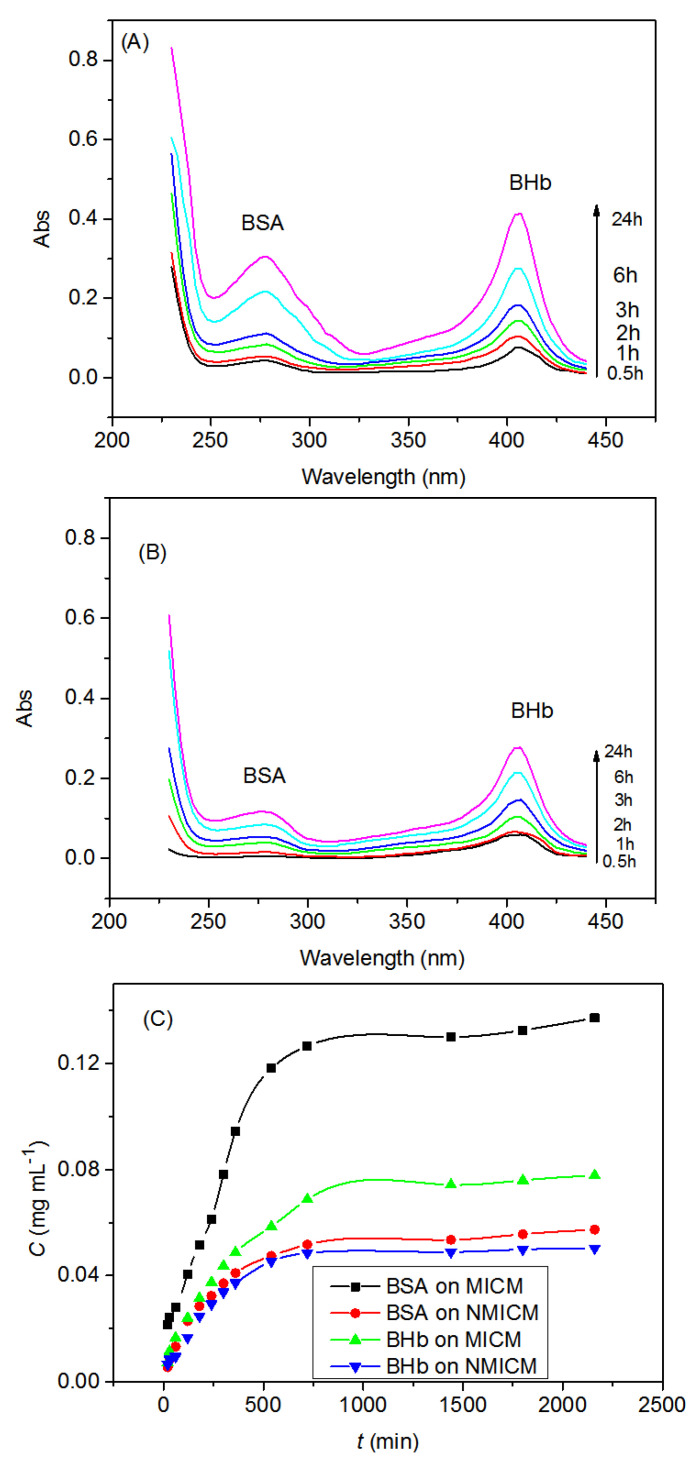
Typical UV-spectra of the BSA/BHb mixed solution in the receptor chamber for MICM (**A**), NMICM (**B**) and the permeation kinetic curves (**C**).

The UV-spectra of the BSA/BHb mixed solution in the receptor chamber for MICM and NMICM were shown in Figure 9A,B, respectively, and the concentrations of BSA and BHb at predetermined time intervals were determined to plot the permeation kinetic curves in Figure 9C. The concentrations of BSA and BHb increased rapidly with the permeation time within 720 min, and then increased slowly. The permeation rate of BSA through MICM was much higher than that of BHb, while the permeation rates of BSA and BHb through NMICM were very approximate. Moreover, the permeation rate of BSA through MICM was much higher than that through NMICM.

In Table 6, the permselectivity factor (*SF*) of MICM was higher than that of NMICM, and the transporting factor (*TF*) of BSA was higher than that of BHb. These results can be explained as follows: the MICM had many imprinted cavities for BSA to transport through the MICM, and the imprinted cavities with the memory of the template molecular in size, spatial structure and functional groups could more specifically recognize BSA than BHb and since NMICM had no specific recognition cavities, the values of *P* of BSA and BHb were very close.

**Figure 10:**
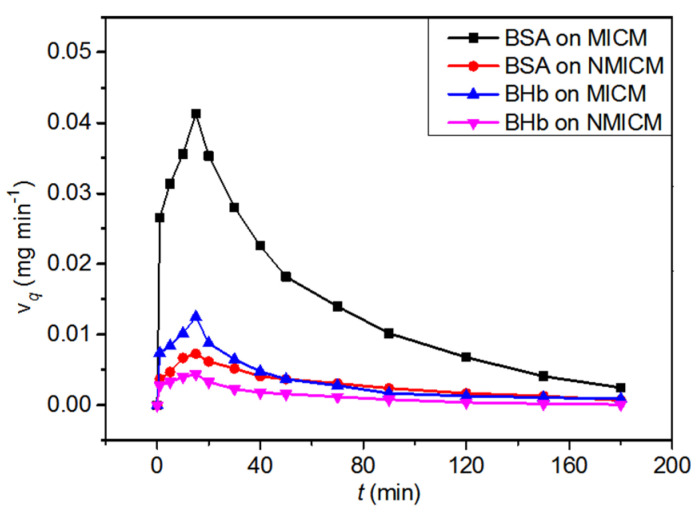
The dynamic adsorption curves of MICM and NMICM in the BSA/BHb mixed solution.

The dead-end membrane filtration experiment was carried out to investigate the dynamic adsorptions of MICM and NMICM in the BSA/BHb mixed solution. The dynamic adsorption curves and adsorption performance were listed in Figure 10 and Table 7, respectively. *Q_d_* of MICM toward BSA was much higher than that of NMICM (*DF* = 4.91), and *Q_d_* of MICM toward BSA was much higher than that toward BHb. Compared with NMICM, MICM had much higher adsorption capacity and dynamic adsorption imprinting factor, because MICM had a lot of imprinted sites which would significantly improve adsorption selectivity toward the imprinted template (BSA) during the adsorption process. Therefore, MICM had a good separation ability than NMICM because of the specific recognition ability of MICM even when BHb, having similar molecular weight to BSA, was present in the protein mixed solution.

### 2.8. Separation Performance of MICM and NMICM in Calf Serum Solution by Solid-Phase Extraction and Membrane Adsorption

**Figure 11:**
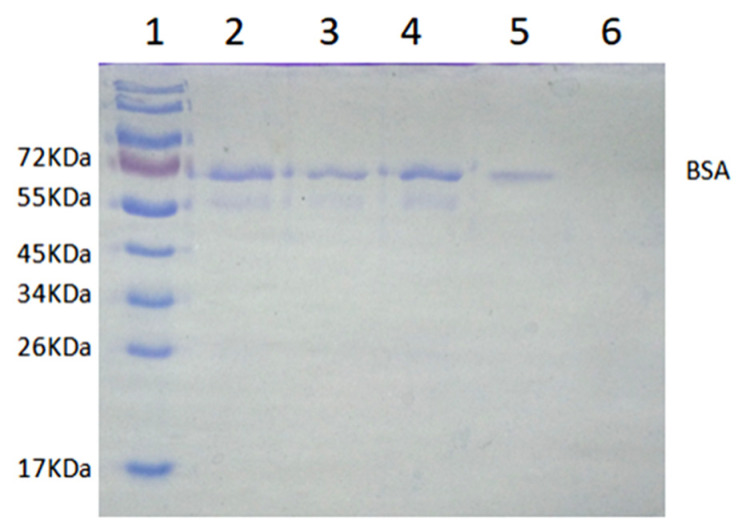
The SDS-PAGE analysis of isolating BSA from calf serum by solid-phase extraction: Lane 1, 10 μL of protein molecular weight marker; lane 2, 10 μL of 100-fold dilution of calf serum solution before adsorption; lane 3, 10 μL of 100-fold dilution calf serum solution after adsorption with the MICM; lane 4, 10 μL of calf serum solution after adsorption with the NMICM; lane 5, 10 μL of the recovery solution from MICM; lane 6, 10 μL of the recovery solution from NMICM.

As shown in Figure 11, the lane of the calf serum sample had a darker band near 66 kDa than other bands, which indicated that BSA was the main component in calf serum. Compared with the band of BSA in lane 2, the band of BSA in lane 3 had a lighter color, while the band of BSA in lane 4 had little change. The results revealed that the MICM had a higher adsorption capacity toward BSA than NMICM. After desorption, only the band of BSA was observed in lane 5, while the band of BSA was hardly observed in lane 6, which suggested that much more BSA was recovered from MICM than from NMICM. The results showed that MICM had better separation performance than NMICM due to the imprinting efficiency.

The SDS-PAGE analysis of isolating BSA from calf serum by membrane adsorption was shown in Figure S1. After desorption from MICM, there was only BSA in the recovery solution (lane 3), while no BSA was observed in the recovery solution from NMICM (lane 4). All these results revealed that the recognition specificity of MICM toward BSA was much higher than that of NMICM.

## 3. Experimental

### 3.1. Materials and Reagents

Bovine serum albumin (BSA, ≥98% mass purity, molecular weight (MW) 67.0 kDa, isoelectric point (pI) 4.8), lysozyme (Lys, ≥90% mass purity, MW 14.4 kDa, pI 11.2), Bovine hemoglobin (BHb, ≥90% mass purity, MW 64.5 kDa, pI 6.8), and Tris-HCl buffer were purchased from Solarbio Science & Technology Co., Ltd., Beijing, China. Glycine (≥99% mass purity) and ovalbumin (OVA, ≥99% mass purity, 45.0 kDa, pI 4.7) were purchased from Sigma Chemical Co., Ltd., Santa Clara, CA, USA. Calf serum was purchased from Pingrui Biotechnology Co., Ltd., Beijing, China. Sodium alginate (SA, G/M = 1.21, viscosity 1.05–1.15 pa·s), N,N-methylene bisacrylamide (MBA, >99.9% purity) and CaCl_2_ (>99% purity) were purchased from Damao Chemical Reagent Factory, Tianjin, China. N,N,N,N-Tetramethylethylenediamine (TEMED, >99% purity), 1-Vinylimidazole and 2-chloroacetamide were purchased from Aladdin Co., Ltd., Shanghai, China. Ammonium persulfate (APS, >98% purity) was purchased from Xilong Chemistry Co., Ltd., Shantou, China. All other chemical agents are analytical grade.

### 3.2. Preparation of Functionalized Ionic Liquid [VAMIM]Cl

The functional monomer [VAMIM]Cl was synthesized according to the method in the literature [18,20]. The chemical structure of the IL was confirmed by NMR which was shown in our previous work [18].

### 3.3. Preparation of MICM

The scheme of the preparation process of the MICM was described in Scheme 1, and a typical synthesis process was as follows. First, functional monomer [VAMIM]Cl (1.2010 g) was added to the PBS solution containing BSA (10 mL, 100 mg mL^−1^), and ultrasonicated in an ice water bath until completely dissolved, and then the cross-linking agent MBA (0.1230 g) was added and ultrasonicated in ice water for 20 min. Second, SA (0.2404 g) was added to the mixture and stirred until no powder was observed in the solution, and then under magnetic stirring, initiator APS (0.5 mL) and catalyst TEMED (0.3 mL) was added to obtain a casting solution. Third, the casting solution (0.9 mL) was poured into a Petri dish 3.5 cm in diameter, and then frozen and polymerized in a refrigerator at −18 °C for 12 h. Fourth, the membrane was immersed in 5% (*w*/*w*) of calcium chloride (CaCl_2_) aqueous solution to ionically crosslink for 2 h. In order to remove the BSA which was used as a template in the membrane, the membrane was then repeatedly washed with 10 mmol L^−1^ of Tris-HCl buffer solution (pH = 7.0) containing 0.8% (*w*/*w*) CaCl_2_ until BSA could not be detected by an ultraviolet spectrometer. Finally, the membrane was freeze-dried, and then the obtained MICM was stored in a desiccator until use. 

The preparation process of non-imprinted cryogel membrane (NMICM) was the same as that of MICM, except for the addition of the template BSA.

### 3.4. Characterization

The surface morphology of the membrane was observed by scanning electron microscope (SEM, JSM-6701F, Jeol Ltd., Tokyo, Japan). The Fourier transform infrared spectroscopy (FTIR) was collected on a Fourier transform infrared spectrometer (Nicolet 5700, Thermo Company, Waltham, MA, USA). The thermal stability of the membrane was studied on a thermogravimetric analyzer (STA2500, Netzsch, Waldkraiburg, Germany) with a heating rate of 10 °C min^−1^ under a dynamic N_2_ atmosphere. The mechanical properties of the membrane were tested using a tensile testing machine (CMT8502, MTS Industrial System Co., Ltd., Shanghai, China).

### 3.5. Measurement of Porosity and Swelling Ratio of the Membranes

The weighing method was used to determine the equilibrium swelling ratio and macropore porosity of the membrane according to the literature [21,26,37]. First, the membrane (0.01 g) was swelled in 0.8% CaCl_2_/10 mmol L^−1^ Tris-HCl buffer solution until reaching a swelling equilibrium at room temperature. Then, the membrane was taken out from the solution; the surface water was removed by a filter paper and the mass (*m_swollen_*, g) of the swollen membrane was measured. Second, the water in the swollen membrane was squeezed out under compression, and the weight of the squeezed membrane (*m_squeezed_*, g) was determined. Third, the squeezed membrane was dried at 60 °C until the weight of the membrane remained unchanged, and then the mass (*m_dry_*, g) of the dry membrane was determined. 

The macropore porosity (*Porosity*) and equilibrium swelling ratio (*ESR*) were calculated by Equations (6) and (7), respectively [21,26,37].

(6) $$Porosity=\frac{m_{swollen}-m_{squeezed}}{m_{squeezed}}\times 100\%$$

(7) $$ESR=\frac{m_{swollen}-m_{dry}}{m_{dry}}\times 100\%$$

### 3.6. Adsorption Experiments

The adsorption amount of the membrane was determined by batch adsorption experiments. A typical adsorption process was as follows. First, the dry membrane (0.01 g) was fully swelled in 0.8% CaCl_2_/10 mmol L^−1^ Tris-HCl buffer solution, and then the membrane was oscillated in 20 mL of protein solution at 25 °C for 10 h. The aim of the addition of 0.8% CaCl_2_ was to avoid the loss of Ca^2+^ ions from the membrane and improve the mechanical property of the membranes. The swelling media in the adsorption process was similar to the preparing media of the gel, which can keep the shape of the imprinted cavity of the MICM to maintain the adsorption specificity. After adsorption, the concentration of protein in the solution after adsorption was determined by UV–vis spectrometry. The detection wavelength for BSA, Lys, OVA and BHb was 278 nm, 280 nm, 280 nm and 405 nm, respectively.

The equilibrium adsorption amount (*Q*) was calculated by Equations (8) and (9) [38].

(8) $$Q_{e}=\frac{(C_{0}-C_{e})V}{m}$$

(9) $$Q_{t}=\frac{(C_{0}-C_{t})V}{m}$$

where *Q_e_* (mg g^−1^) and *Q_t_* (mg g^−1^) are the amount of the protein-bound on the membrane at equilibrium and time *t*, respectively; *C*_0_ (mg mL^−1^), *C_e_* (mg mL^−1^) and *C_t_* (mg mL^−1^) are the concentrations of protein initially, at equilibrium and time *t*, respectively; *V* (mL) is the volume of the sample solvent; *m* (g) is the mass of the membrane used.

In addition, the imprinting factor (*IF*) and selectivity coefficient (*α*) can evaluate the selectivity of the MICM, which can be calculated by Equations (10) and (11), respectively [38,39].

(10) $$IF=\frac{Q_{MICM}}{Q_{NMICM}}$$

(11) $$\alpha =\frac{IF_{tem}}{IF_{ref}}$$

where *Q_MICM_* and *Q_NMICM_* represent the adsorption amount of MICM and NMICM, respectively; *IF_tem_* and *IF_ref_* are the imprinting factors toward the template protein (BSA) and reference protein (Lys, OVA or BHb), respectively.

### 3.7. Membrane Permeation and Dead-End Filtration Experiment

**Figure 12:**
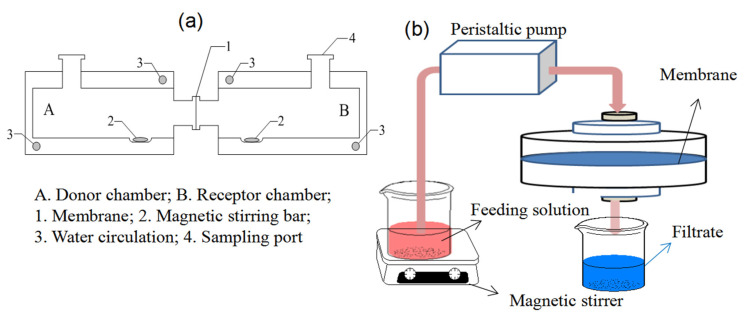
The membrane permeation (**a**) and dead-end filtration experimental (**b**) setups.

The Valia–Chien permeation cells shown in Figure 12a were used to carry out the permeation experiments, which consisted of the donor chamber and receptor chamber. The cross-section diameter of the opening of the permeation cells was 1.0 cm. The membrane was mounted onto the opening between the two chambers. The feeding solution (15 mL) in the donor chamber contained BSA and BHb with equal initial concentrations of 0.3 mg mL^−1^, and blank 0.8% CaCl_2_/10 mmol L^−1^ Tris-HCl buffer solution (15 mL) was added into the receptor chamber. The solutions were electromagnetically stirred at 25 °C. The protein concentration (*C_R_*) in the receptor solution was analyzed by UV-spectra at a predetermined time interval, and the permeation amount of the protein (*P*), transporting factor (*TF*) and permselectivity factor (*SF*) can be calculated by Equations (12)–(14), respectively [39].

(12) $$P=\frac{C_{R}V_{R}}{A}$$

(13) $$TF=\frac{P_{MICM}}{P_{NMICM}}$$

(14) $$SF=\frac{P_{tem}}{P_{ref}}$$

where *P* (mg cm^−2^) is the amount of the protein permeated through the membrane; *C_R_* (mg mL^−1^) represents the protein concentration in the receptor solution; *V_R_* (mL) is the volume of the receptor solution; *A* (cm^2^) is the valid surface area of the membrane; *P_MICM_* and *P_NMICM_* are the permeation amounts of the protein transported through MICM and NMICM, respectively; *P_tem_* and *P_ref_* are the permeation amounts of the template protein and reference protein, respectively.

In Figure 12b, the dynamic adsorptions were performed on the dead-end filtration apparatus. The BSA/BHb mixed solution (0.2 mg mL^−1^ for each protein) at 25 °C was filtrated through the membrane at 1.25 mL min^−1^ of the flow rate by a peristaltic pump, and protein concentration in the filtrates was measured at a predetermined time interval. The adsorption capacity per unit area of the membrane *Q_d_* (mg cm^−2^), total adsorption capacity of the membrane *Q_T_* (mg) and dynamic adsorption imprinting factor (*DF*) can be calculated by Equations (15)–(17), respectively [38].

(15) $$v_{q}=\left(C_{i}-C_{f}\right)v$$

(16) $$Q_{d}=\frac{Q_{T}}{A}$$

(17) $$DF=\frac{Q_{d,MICM}}{Q_{d,NMICM}}$$

where *v_q_* (mg min^−1^) is the adsorption rate on the membrane at a different time; *C_i_* (mg mL^−1^) is the protein concentration in the feeding solution; *C_f_* (μmol mL^−1^) is the protein concentration in the filtrate at a different time; *v* (mL min^−1^) is the flow rate of the feeding solution; *Q_T_* (mg) is the total adsorption capacity of the membrane, which is equal to the area under *v_q_*-*t* curve; *A* (cm^2^) is an effective area of the membrane; *Q_d,MICM_* and *Q_d,NMICM_* (mg cm^−2^) are *Q_d_* of MICM and NMICM, respectively.

### 3.8. Reusability Experiment

To evaluate the reusability of the membrane, five cycles of adsorption–desorption were performed. The dry membrane (0.01 g) was first swollen in 0.8% CaCl_2_/10 mmol L^−1^ Tris-HCl buffer solution, and then oscillated in 20 mL of 0.5 mg mL^−1^ BSA solution at 25 °C for 6 h. Next, the membrane was taken out from the protein solution and washed by 0.8% CaCl_2_/10 mmol L^−1^ Tris-HCl buffer solution repeatedly until no protein was detected in the elution. After that, the washed membrane was reused for another adsorption–desorption cycle. The adsorption–desorption was repeated five times, and *Q_e_* in each cycle was measured.

### 3.9. Separation Performance of MICM and NMICM in Calf Serum Solution by Solid-Phase Extraction and Membrane Adsorption

The separation performance of membranes was tested by a solid-phase extraction experiment. First, the membrane (0.01 g) was incubated in 20 mL of 100-fold dilution of calf serum solution for 6 h, and then the membrane was collected by centrifugation. Second, the membrane was washed rapidly with 0.8% CaCl_2_/Tris-HCl solution (4 mL) to remove protein from its surface, and then incubated in 0.8% CaCl_2_/Tris-HCl solution (5 mL) to recover protein from the membrane. Finally, the initial calf serum solution, the calf serum solution after adsorption and the recovery solution were analyzed by sodium dodecyl sulfate polyacrylamide gel electrophoresis (SDS-PAGE). 

The membrane adsorption experiment was also used to examine the separation performance of membranes. The 80-fold dilution of calf serum solution was filtrated through the membrane at a flow rate of 1.25 mL min^−1^ by the dead-end filtration. After 3 h, the membrane was washed rapidly with 0.8% CaCl_2_/Tris-HCl solution (4 mL) to remove protein from the surface of the membrane, and then incubated in 0.8% CaCl_2_/Tris-HCl solution (5 mL) to recover protein from the membrane. The initial calf serum solution and the recovery solution were analyzed by SDS-PAGE. 

## 4. Conclusions

In this work, the method combining the composite structure and imprinting technology was applied to enhance the strength of poly(IL)-based BSA-imprinted cryogel membrane and maintain a good separation efficiency at the same time. The results showed that the tensile strength and elongation of the MICM were 30.30 and 8.28 times higher than the poly(IL) membrane, respectively. Moreover, the MICM had a very high adsorption capacity of 485.87 mg g^−1^ at 20 °C and 0.66 mg mL^−1^ of initial concentration, and also, showed good imprinting efficiency toward BSA. We believe that the construction strategy of this high-strength BSA-imprinted cryogel membrane will provide a new method for preparing a novel protein-imprinted cryogel membrane possessing both high mechanical strength and good separation performance. Following the cross-linking of SA and Ca^2+^, the formed CA significantly improved the mechanical properties of the obtained membrane, further investigation deserves on employing the CA to mediate polymer mechanical strength.

## Figures and Tables

**Table 1:** Kinetic parameters for the pseudo-first-order and the pseudo-second-order kinetic models.

Membranes	Pseudo-First-Order Kinetic Model			Pseudo-Second-Order Kinetic Model		
	*Q_e,cal_* [mg g^−1^]	*k*_1_ [min^−1^]	R^2^	*Q_e,cal_* [mg g^−1^]	*k*_2_ [g mg^−1^ min^−1^]	R^2^
MICM	298.41	0.02179	0.9960	337.72	8.21 × 10^−5^	0.9853
NMICM	139.78	0.01361	0.9941	165.63	9.38 × 10^−5^	0.9875

**Table 2:** Parameters of isotherm at various temperatures.

Membranes	*T* [°C]	Langmuir Mode				Freundlich Mode		
		*K_L_* [mL mg^−1^]	*Q_max_* [mg g^−1^]	*R_L_*	R^2^	1/*n*	*K_F_*	R^2^
MICM	20	6.788	606.3	0.141~0.596	0.9906	0.3969	617.7	0.9181
	25	3.704	593.6	0.231~0.730	0.9960	0.4923	535.7	0.9613
	30	2.449	582.3	0.312~0.803	0.9974	0.5674	467.0	0.9741
NMICM	20	3.445	294.2	0.244~0.744	0.9945	0.4741	251.3	0.9554
	25	2.527	282.3	0.305~0.798	0.9882	0.5292	220.5	0.9533
	30	1.938	275.7	0.364~0.837	0.9757	0.5849	197.2	0.9408

**Table 3:** The recognition selectivity of MICM and NMICM toward different proteins.

Proteins	*Q_e_* of MICM [mg g^−1^]	*Q_e_* of NMICM [mg g^−1^]	*IF*	*α*
BSA	318.11	147.25	2.16	
OVA	171.13	140.43	1.22	1.77
BHb	136.26	129.26	1.05	2.04
Lys	34.41	19.72	1.74	1.23

**Table 4:** The competitive recognition of MICM and NMICM toward BSA and BHb in the protein mixture solution.

Proteins	*Q_e_* of MICM [mg g^−1^]	*Q_e_* of NMICM [mg g^−1^]	*IF*	*α*
BSA	308.11	151.27	2.04	1.90
BHb	142.97	133.69	1.07	

**Table 5:** A comparison of the maximum adsorption capacity of different adsorbents for BSA.

Adsorbents	Main Interactions	*T* [k]	*Q_max_* [mg g^−1^]	Refs.
MICM	Ion-pair interaction, hydrogen bond and *π-π* interaction.	298.15	593.6	This work
Fe_3_O_4_@MnO_2_@BSA-MIPs	Hydrophobic interaction, hydrogen bonding and other interactions.	298.15	217.39	[33]
Surface imprinted Fe_3_O_4_@rGO microspheres	Hydrophobic interaction, hydrogen bonding and other interactions.	298.15	350.28	[34]
Magnetic chitosan/PVA/laponite RD beads	Electrostatic interaction	298.15	240.5	[35]
Hydroxylated carbon nanotubes	Covalent bonding	298.15	238.2	[36]
*Q_max_* (mg g^−1^) is the theoretical maximum adsorption amount calculated from Langmuir mode				

**Table 6:** The permselectivity of MICM and NMICM in mixture solution of BSA and BHb.

Proteins	MICM		NMICM		*TF*
	*P* [mg cm^−^^2^]	*SF*	*P* [mg cm^−^^2^]	*SF*	
BSA	2.62	1.77	1.09	1.14	2.40
BHb	1.48		0.96		1.54

**Table 7:** The dynamic adsorption performance of MICM and NMICM in the BSA/BHb mixed solution.

Proteins	MICM	NMICM	*DF*	*β*
	*Q_d_* [mg cm^−2^]	*Q_d_* [mg cm^−2^]		
BSA	2.53	0.52	4.91	1.47
Hb	0.73	0.22	3.34	

## Supplementary Materials

The following supporting information can be downloaded at: https://www.mdpi.com/article/10.3390/molecules27217304/s1, Table S1. Preparation parameters and *Qe* of MICM for BSA, *IF*, tensile strength, and breaking elongation. Figure S1. The SDS-PAGE analysis of the isolation of BSA from calf serum by membrane adsorption.

## Sample Availability

Samples of the compounds are not available from authors.
