# Supplementary material for: Preparation and Characterization of Protein Molecularly Imprinted Poly (Ionic Liquid)/Calcium Alginate Composite Cryogel Membrane with High Mechanical Strength for the Separation of Bovine Serum Albumin
Source: Molecules. 2022 Oct 27;27(21):7304. doi: 10.3390/molecules27217304 (PMC9654497; doi:10.3390/molecules27217304)
Supplement: Supplementary file 1 [file molecules-27-07304-s001.zip › molecules-1975890-supplementary.pdf]

Supplementary Information

Preparation and Characterization of Protein Molecularly Imprinted Poly (Ionic Liquid)/Calcium Alginate Composite Cryogel Membrane with High Mechanical Strength for the Separation of Bovine Serum Albumin

Jie-Ping Fan [1,*], Wen-Ya Dong [1], Xue-Hong Zhang [2], Jia-Xin Yu [1], Cong-Bo Huang [1], Li-Juan Deng [3], Hui-Ping Chen [1] and Hai-Long Peng [1]

1 Department of Chemical Engineering, Nanchang University, Nanchang 330031, China;
2 School of Foreign Language, Nanchang University, Nanchang 330031, China
3 School of Chemical Engineering, Ningbo University of Technology, Ningbo 315016, China
* Correspondence: jasperfan@ncu.edu.cn

Table S1. Preparation parameters and Q_e of MICM for BSA, *IF*, tensile strength, and breaking elongation

Membranes[a]	SA [g]	[VAMIM]Cl [g]	MBA [g]	Q_e [mg g^{-1}]	*IF*	Tensile Strength [KPa]	Breaking elongation [%]
MICM0	0	1.2010	0.1230	349.95	2.370	2.97	11.31
MICM1	0.1202	1.2010	0.1230	313.8	0.983	12.63	15.40
MICM2	0.1803	1.2010	0.1230	288.5	1.137	18.15	17.88
MICM3	0.2404	1.2010	0.1230	247.7	1.492	29.62	23.81
MICM4	0.3005	1.2010	0.1230	152.5	1.269	54.25	25.01
MICM5	0.2404	1.2010	0.0615	154.6	1.091	26.90	30.83
MICM6	0.2404	1.2010	0.1845	135.4	0.965	33.36	20.44
MICM7	0.2404	0.8013	0.0820	276.8	1.965	114.00	81.90
MICM8	0.2404	0.6010	0.0615	318.1	2.160	90.00	93.70

[a] All MICMs or NMICMs are prepared by adding the same amount of BSA (0.1 g), Tris-HCl buffer solution (10mL), 0.1 g mL^{-1} APS (0.5 mL) and 0.05 g mL^{-1}TEMED (0.3 mL).

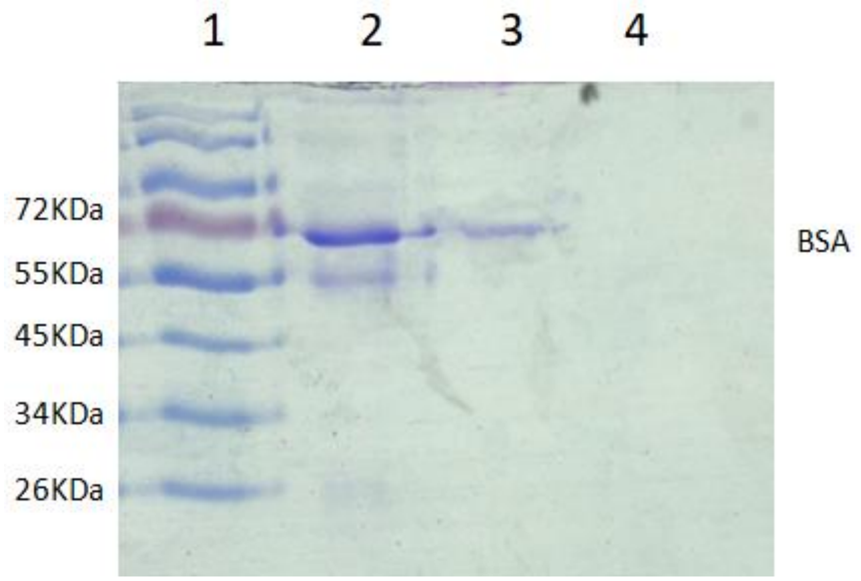

Figure S1. The SDS-PAGE analysis of the isolation of BSA from calf serum by membrane adsorption. Lane 1, 10 μL of protein molecular weight marker; lane 2, 10 μL of 80-fold dilution of calf serum solution before adsorption; lane 3, 10 μL of the recovery solution from MICM; lane 4, 10 μL of the recovery solution from NMICM.
